# Predominant approaches to measuring pregnancy-related anxiety in Sub-saharan Africa: a scoping review

**DOI:** 10.1186/s12889-024-19935-3

**Published:** 2024-09-06

**Authors:** Sophia Dane Fraga, Ibrahim Nawaz Khan, Tanvi A. Sharma, Emma R. Lawrence

**Affiliations:** 1 University of Michigan Medical School, 1301 Catherine St, Ann Arbor, MI 48109 USA; 2 Department of Obstetrics and Gynecology, University of Michigan, 1500 E. Medical Center Dr, Ann Arbor, MI 48109 USA

**Keywords:** Pregnancy, Anxiety, Sub-saharan Africa, Scales, Measurements

## Abstract

**Background:**

Pregnancy-related anxiety significantly impacts maternal and fetal health in low- and middle-income countries (LMICs), including those within Sub-Saharan Africa (SSA). Most studies conducted to evaluate pregnancy-related anxiety in LMICs have utilized scales developed in high-income countries, despite significant variations in pregnancy-related anxiety due to socioeconomic and cultural contexts. This review surveyed existing literature in order to identify which scales have been used to measure pregnancy-related anxiety in SSA.

**Methods:**

A systematic search was conducted in PubMed, Health and Psychosocial Instruments, and APA PsycNet for relevant studies published in the English language up to March 22, 2023. Eligible studies focused on anxiety in pregnant populations within SSA, using validated scales or tools. Screening followed PRIMSA guidelines, with blinded review at the abstract/title level and subsequent full-text review. Data was extracted and analyzed to identify trends and characteristics of the screening tools used.

**Results:**

From 271 articles, 37 met inclusion criteria, identifying 24 different tools used to measure anxiety in pregnant women in SSA. The most common tools were the Generalized Anxiety Disorder 7-item scale (seven uses), State-Trait Anxiety Inventory (five uses), and the Self-Reporting Questionnaire 20 (five uses). Seven tools were pregnancy-specific, with only two designed specifically for SSA: the Risk Factor Assessment (RFA), and the 4-Item Screening Tool. Studies were most frequently conducted in South Africa, followed by Tanzania, Ethiopia, Nigeria, and Ghana.

**Conclusions:**

This scoping review illustrates that only two tools (the RFA and 4-item Screening Tool) were created to assess pregnancy-related anxiety specifically in SSA. This highlights the need for more culturally sensitive tools tailored to the specific contexts of pregnant populations in SSA.

## Background

Pregnancy-related anxiety is defined as worry associated specifically with maternal or infant outcomes, making it distinct from generalized anxiety disorder (GAD) [1]. In fact, studies have shown that most women with anxiety in pregnancy had worries not associated with GAD [1]. A concept analysis broke pregnancy-related anxiety into nine cognitive dimensions: anxiety around fetal health, loss of fetus, childbirth, mother’s wellbeing, body image, parenting and care for child, general health care, financial, and family/social support [2].

Pregnancy-related anxiety is a pervasive concern that affects pregnant individuals worldwide across income levels and geographic locations. Globally, approximately 15.2% of pregnant women meet criteria for an anxiety disorder [3]. Untreated maternal anxiety or depression may increase risk for adverse pregnancy outcomes such as gestational diabetes mellitus, fetal growth restriction, preterm birth, or fetal demise by nearly 3.5 times [4]. Moreover, recent studies have demonstrated a strong association between maternal anxiety and adverse socioemotional, cognitive, motor, and behavioral outcomes in their children. These negative developmental consequences can extend beyond infancy and have enduring effects throughout childhood and adolescence [5].

In low- and middle-income countries (LMICs), the prevalence of anxiety symptoms during pregnancy is even higher than in high-income countries, with reports suggesting that one in four women experience anxiety symptoms [4]. In LMICs, pregnancy-related anxiety represents a particularly significant burden due to the numerous challenges faced by pregnant individuals, including limited access to healthcare, poverty, and social disparities [6]. Sub-Saharan Africa (SSA) notably contains LMICs with the highest rates of maternal morbidity and mortality [7]. Risk factors for development of maternal mood disorders (poverty, food insecurity, intimate partner violence, and comorbid medical conditions) are common in many countries within SSA [8]. However, research on prevalence of pregnancy-related anxiety in SSA has been limited [8, 9].

Determining the prevalence of pregnancy-related anxiety depends on the adaptation and execution of validated scales to measure anxiety. Most of the existing research conducted in SSA settings has relied on scales and measures developed in high-income countries. However, scales developed in high-income countries may not adequately capture the unique determinants of pregnancy-related anxiety in SSA [10–12]. Additionally, in applying these scales, users may encounter barriers such as translation errors, administrative challenges in limited-resource settings, or overly complex and inaccessible language [10, 13]. Furthermore, the concerns of pregnant women and clinical presentation of pregnancy-related anxiety in SSA may be distinct due to cultural differences [10, 14, 15]. This highlights the need for culturally and contextually sensitive scales that are specifically validated for use in SSA populations. Site-specific validated scales are needed to quantify the overall burden of pregnancy-related anxiety, evaluate individual patients in clinical environments, and accurately assess the efficacy and impact of mental health interventions [16].

To address these critical gaps, we undertook a scoping review of validated screening tools for detecting pregnancy-related anxiety in SSA, with a focus on identifying scales validated within SSA contexts. Throughout this review, we aimed to (1) describe the use of pregnancy-related anxiety scales evaluated in the literature by type of scale and study setting, and (2) summarize characteristics of the scales, including length and question type, that may impact the practicality of administration in low-resource settings.

## Methods

This scoping review was conducted following the recommendations from the Preferred Reporting Items for Systematic Reviews and Meta-Analysis (PRISMA) [17] and adhered to the methodological framework first outlined by Arksey & O’Malley [18].

The primary aim of this scoping review was to identify and determine the current screening tools utilized to assess pregnancy-related anxiety in SSA. The scope of this project was intentionally limited and not intended to serve as a comprehensive synthesis or systematic review of all relevant literature on pregnancy-related anxiety in SSA. As such, it provides an overview of the tools and methodologies applied in existing studies rather than an exhaustive analysis.

### Information sources, search strategy, and eligibility criteria

**Fig. 1:**
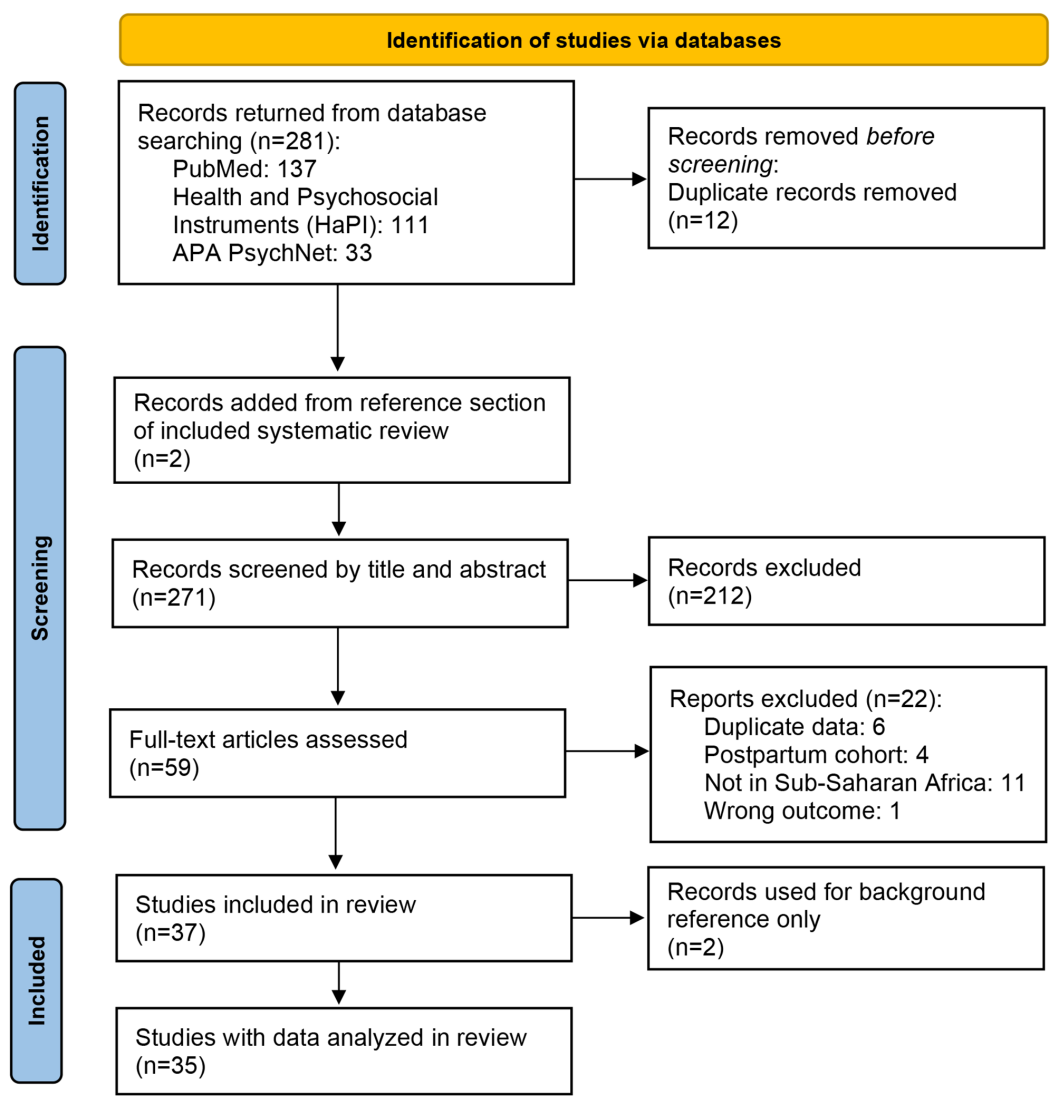
PRISMA flow chart of study selection

A comprehensive search strategy was developed by the research team, which consisted of an obstetrician, research assistants, medical students, and an experienced health sciences librarian. The databases PubMed, Health and Psychosocial Instruments (HaPI), and APA PsycNet were searched systematically for relevant studies in the English language that assessed pregnancy-related anxiety in SSA using validated scales. All searches were conducted on March 22, 2023 and included any relevant studies published up to that date. The search terms encompassed concepts related to pregnancy, anxiety, Sub-Saharan Africa, and tool evaluation (Table 1). The search strategy adhered to the PRISMA guidelines, with Fig. 1 outlining the search refinement process.

**Table 1:** Search strategy terms

Set	Search
Pregnancy	“Pregnancy”[MeSH] OR “pregnan”*[tiab]”
Anxiety	“Anxiety”[MeSH] OR “anxi*[tiab]” OR “worry*[tiab]”
Africa	“Africa South of the Sahara”[MeSH] OR “Africa, western”[MeSH] “
Tool Evaluation	“Surveys and Questionnaires”[MeSH] OR “Patient Health Questionnaire”[MeSH] OR “Test Anxiety Scale”[MeSH] OR “measur*[tiab]” OR “scale*[tiab]”

The initial database search identified 281 records, from which 12 duplicate articles were removed, yielding 269 unique records. These were exported to Rayyan, a web-based tool designed for reviews. An additional two articles were identified through reference list reviews, bringing the total to 271 articles for screening. Abstracts and titles were reviewed blindly by three independent researchers (SF, TS, IK) using inclusion criteria and exclusion criteria, as detailed below. Blinding limited bias by preventing reviewers from seeing each other’s decisions until all articles had been independently reviewed. Articles were included only if all three readers agreed on inclusion, and discrepancies were resolved via consensus discussions.

From the 271 article abstracts screened for inclusion, 59 met the initial criteria and were subjected to full-text review. Of these, 37 articles were deemed relevant for data extraction, with exclusions made for reasons such as duplicate data, focus on postpartum cohorts, or research conducted outside of SSA, as detailed in the PRISMA diagram (Fig. 1).

Eligible studies were required to meet the following criteria: (1) focus on anxiety screening, (2) examination of anxiety specifically among pregnant women, (3) conduct of research in Sub-Saharan Africa, and (4) use of a specific, validated scale or tool for evaluating pregnancy-related anxiety. Studies with a primary focus on other psychological conditions (e.g., depression, psychosis) were included only if they provided data on pregnancy-related anxiety measures.

During the search process, it was noted that many scales utilized were not specific to pregnancy-related anxiety but were originally developed for GAD. Despite the distinct nature and presentation of pregnancy-related anxiety [1], these generalized scales were included to reflect historical and current practices in the assessment of anxiety during pregnancy. Additionally, studies evaluating peripartum “common mental disorders,” as previously defined by the World Health Organization [19], were included in the review.

Exclusion criteria included (1) studies that utilized qualitative methods, such as narrative descriptions, (2) studies that utilized focus groups, (3) studies that assessed pregnancy-related anxiety in retrospect, and (4) unpublished studies or studies pending publication.

### Data extraction

Data was extracted and reviewed by three authors (SF, IK, TS). Each full text article was examined for the country of study implementation, the number of participants, participant age range, the screening tool(s) used, and the outcome examined (e.g., common mental disorder vs. anxiety) (Table 2). In cases where multiple screening tools were used to evaluate different outcomes, only data pertaining to anxiety measurement were extracted; scales that exclusively measured another mental health disorder without components of anxiety (e.g., depression, psychosis) were excluded. An exception was made for three studies that utilized the Edinburgh Postnatal Depression Scale (EPDS) to measure anxiety symptoms in pregnant women [20–22]. No additional studies were identified for inclusion upon further review of references.

**Table 2:** Studies included in review on pregnancy-related anxiety in Sub-saharan Africa

Author (Year)	Scale	Outcome Examined	*N*	Age Range, years	Country
Van Heyningen (2018) [20]	EPDS; Kessler Psychological Distress Scale (K10), GAD-2, Whooley Questions	Common Perinatal Mental Disorders (CPMD)	376	18–48	South Africa
Vythilingum (2013) [21]	EPDS and Risk Factor Assessment (RFA)	Psychological Distress	1460	Mean 25.26	South Africa
Abrahams (2019) [22]	EPDS, 4-Item Screening tool	Common Mental Disorders	66	15–38	South Africa
Umuziga (2020) [23]	Zung Self-rating Anxiety Scale (SAS)	Anxiety	165	≥ 15	Rwanda
Abiodun (1994) [24]	Hospital Anxiety and Depression Scale (HADS)	Anxiety and Depression	240	N/A	Nigeria
Redinger (2020) [25]	State Trait Anxiety Index (STAI)	Anxiety	649	25–34	South Africa
Rwakarema (2015) [26]	Pregnancy-Related Anxiety 10-item tool (PRAQ)	Anxiety	397	18–42	Tanzania
Bindt (2012) [27]	Generalized Anxiety Disorder 7-item scale (GAD-7); World Health Organization Disability Assessment Schedule 2.0 (WHODAS 2.0)	Anxiety and Perceived Disability	1030	18–46	Ghana and Ivory Coast
Shuffrey (2021) [28]	STAI	Anxiety	274	NA	South Africa
Khwepeya (2018) [29]	Wijma Delivery Expectancy/Experience Questionnaire Version A (W-DEQ-A)	Childbirth Fear	152	26	Malawi
Sanfilippo (2020) [30]	Self-Reporting Questionnaire 20 (SRQ-20)	Common Mental Disorders	124	18–40	The Gambia
Bante (2021) [31]	GAD-7	Comorbid Anxiety and Depression	676	Mean 27.4	Ethiopia
Ngocho (2019) [32]	Brief Symptoms Index (BSI-18)	Anxiety	200	25–35	Tanzania
Fatoye (2004) [33]	STAI	Anxiety	156	N/A	Nigeria
Aderibigbe (1996) [34]	General Health Questionnaire 28 (GHQ-28); anxiety and insomnia are evaluated using items 8–14	Anxiety and Insomnia	277	N/A	Nigeria
Wall (2018) [35]	10-item PRA Questionnaire (PRA-Q)	Anxiety	212	16–44	Tanzania
Gelaw (2020) [37]	Wijma Delivery Expectancy/Experience Questionnaire (W-DEQ)	Fear of Childbirth	387	18–42	Ethiopia
Redinger (2018) [37]	STAI	Anxiety	946	NA	South Africa
Pobee (2022) [38]	Beck Anxiety Inventory (BAI)	Anxiety	116	18–38	Ghana
Hanlon (2009) [39]	SRQ-20	Common Mental Disorders (anxiety, depression, panic, somatic symptoms)	1065	15–49	Ethiopia
Yamamoto (2019) [40]	Pregnancy-related Anxiety Scale-revised (PRaS-r) and Perceived Stress Scale (PSS)	Stress	227	22–30	Tanzania
Barthel (2016) [4141]	GAD-7	Anxiety	778	18–46	Ghana (*n* = 289) and Ivory Coast (*n* = 489)
Say (2016) [42]	GAD-7	Anxiety	250	≥ 15	Malawi, Kenya
Purcell-Jones (2019) [43]	Numerical Visual Analog Anxiety Scale	Anxiety	175	Mean 32.1	South Africa
Roos (2013) [44]	K10 + Spielberger State Inventory (SSI)	Distress (K10) and Anxiety (SSI)	105	Mean 25	South Africa
Kugbey (2021) [45]	HADS	Anxiety and Depression	214	≥ 18	Ghana
Nwafor (2021) [46]	Depression Anxiety Stress Scale-21 (DASS-21)	Psychological Morbidities (depression, anxiety, and stress)	456	18–45	Nigeria
Woldetsadik (2019) [47]	SRQ-20	Common Mental Disorders	743	Mean 27.3	Ethiopia
Ukpong (2004) [48]	GHQ-30, STAI	Psychiatric Morbidity	55	20–44	Nigeria
Spedding (2020) [49]	SRQ-20	Common Mental Disorders (Psychological Distress)	664	N/A	South Africa
Anbesaw (2021) [50]	DASS-21	Anxiety	423	18–38	Ethiopia
Mahenge (2015) [51]	Hopkins Symptom Checklist	Anxiety and Depression	1180	N/A	Tanzania
Masiano (2022) [52]	SRQ-20	Common Mental Disorders	798	Mean 27.5	Malawi
Mashegoane (2021) [53]	Tilburg Pregnancy Distress Scale (TPDS)	Pregnancy-Specific Psychological Distress	205	Mean 27.69	South Africa
Spies (2009) [54]	K10	DSM-IV mood and anxiety disorders	129	15–43	South Africa

The final stage of data extraction was performed on each full-length pregnancy anxiety screening tool, to examine the characteristics of each tool. Reviewers obtained data on the pregnancy-specificity of each tool, as well as the number of questions, types of questions, and number of answer options (Table 3).

**Table 3:** Screening tools assessing pregnancy-related anxiety in Sub-saharan Africa

Scale	Countries Where Used (Number of Studies)	Specific to Pregnancy?	Number of Questions	Type of Question	Number of Answer Choices
Pregnancy-Related Anxiety Questionnaire 10-item tool (PRAQ)	Tanzania (2)	Yes	10	Likert	4
Edinburgh Postnatal Depression Scale (EPDS)	South Africa (3)	Yes	10	Likert	4
Pregnancy-related Anxiety Scale-revised (PrAS-r)	Tanzania	Yes	33	Likert	4
Wijma Delivery Expectancy/Experience Questionnaire Version A (W-DEQ-A)	Malawi, Ethiopia	Yes	33	Likert	6
Risk Factor Assessment (RFA)^a^	South Africa	Yes	11	Yes/No	2
Tilburg Pregnancy Distress Scale (TPDS)	South Africa	Yes	16	Likert	4
4-Item Screening Tool	South Africa	Yes	4	Yes/No	2
Zung Self-rating Anxiety Scale (SAS)	Rwanda	No	20	Likert	4
Hospital Anxiety and Depression Scale (HADS)^b^	Nigeria, Ghana	No	14^c^	Likert	4
State Trait Anxiety Index (STAI)	South Africa (3), Nigeria (2)	No	40	Likert	4
Generalized Anxiety Disorder Assessment 7-item scale (GAD-7)	Côte d’Ivoire (2), Ghana (2), Ethiopia, Malawi, Kenya	No	7 (+ 1)^d^	Likert	4
World Health Organization Disability Assessment Schedule 2.0 (WHODAS 2.0)	Côte d’Ivoire, Ghana	No	12	Likert	5
Self-Reporting Questionnaire 20 (SRQ-20)	The Gambia, Ethiopia (2), South Africa, Malawi	No	20	Yes/No	2
Kessler Psychological Distress Scale (K10)	South Africa (3)	No	10	Likert	5
GAD-2	South Africa	No	2	Likert	4
Whooley Questions	South Africa	No	2	Yes/ No	2
Brief Symptoms Index (BSI-18)^b^	Tanzania	No	6^e^	Likert	6
GHQ-28 (items 8–14)	Nigeria	No	28	Likert	4
Beck Anxiety Inventory (BAI)	Ghana	No	21	Likert	4
Perceived Stress Scale (PSS)	Tanzania	No	10	Likert	5
Numerical Visual Analog Anxiety Scale	South Africa	No	1	Number Line	10
Depression Anxiety Stress Scale-21 (DASS-21)^b^	Nigeria Ethiopia	No	21	Likert	4
GHQ-30	Nigeria	No	30	Likert	4
Hopkins Symptom Checklist^b^	Tanzania	No	25	Likert	4

^a^Scale screens for symptoms/risk factors not related to anxiety

^b^Scale screens for other psychological conditions in addition to anxiety

^c^Only 7 questions in questionnaire screen for anxiety

^d^Anxiety subscale is 6 questions

^e^Additional question at the end of scale that asks the degree of symptom impact on daily life activities

### Data synthesis

Extracted data were analyzed to identify trends in the use of screening tools across SSA, including country-specific usage patterns. First, data for each pregnancy-related anxiety screening tool was analyzed. Geographic data was compiled, examining the different countries each scale was used in and the number of instances of use in each country. This data was then synthesized along with the data extracted regarding scale characteristics (pregnancy specificity, number of questions, type of scale, number of question options), providing a comprehensive overview of the usage and characteristics of each screening tool (Table 3).

Data was then analyzed by country, examining which scales were used in each country, and in how many instances they were used (Table 4).

**Table 4:** Scales used to evaluate pregnancy-related anxiety by country

Country	Scales Used^a^
Ghana	Hospital Anxiety and Depression Scale (HADS) Generalized Anxiety Disorder Assessment 7-item scale (GAD-7) – 2 studies World Health Organization Disability Assessment Schedule 2.0 (WHODAS 2.0) Beck Anxiety Inventory (BAI)
Cote d’Ivoire	GAD-7–2 studies WHODAS 2.0
Ethiopia	GAD-7 Wijma Delivery Expectancy/Experience Questionnaire Version A (W-DEQ-A) Self-Reporting Questionnaire 20 (SRQ-20) – 2 studies Depression Anxiety Stress Scale-21 (DASS-21)
The Gambia	SRQ-20
Kenya	GAD-7
Malawi	GAD-7 W-DEQ-A SRQ-20
Nigeria	State-Trait Anxiety Inventory (STAI) – 2 studies GHQ-28 (items 8–14) DASS-21 GHQ-30
Rwanda	Zung Self-rating Anxiety Scale (SAS)
South Africa	SAI – 3 studies SRQ-20 Edinburgh Postnatal Depression Scale (EPDS) – 3 studies Kessler Psychological Distress Scale (K10) – 3 studies GAD-2 Whooley Questions Numerical Visual Analog Anxiety Scale Risk Factor Assessment (RFA) Tilburg Pregnancy Distress Scale (TPDS) 4-item Screening Tool
Tanzania	Pregnancy-Related Anxiety Questionnaire 10-item tool (PRAQ) – 2 studies Brief Symptoms Index (BSI-18) Pregnancy-related Anxiety Scale-revised (PraS-r) Perceived Stress Scale (PSS) Hopkins Symptom Checklist

^a^Each scale used once per country unless otherwise noted

## Results

### Study selection

The study selection is outlined in the PRISMA diagram (Fig. 1). All the included studies assessed pregnancy-related anxiety, with individual studies doing so at different points across all trimesters. Various methods were used to quantify levels of anxiety, with some studies having cut-off scores and others providing ranges to estimate anxiety.

### Study characteristics

Thirty-five full-text articles contained studies that used a scale to evaluate mental health in pregnant women [20–54] (Table 2). The specific outcomes evaluated in the studies varied slightly, but all studies were focused on evaluating forms of anxiety during pregnancy. Of the 35 studies, the most common outcome examined was anxiety (13 studies), followed by common mental disorders (seven studies), anxiety and depression (four studies), psychological distress (three studies), and childbirth fear (two studies) (Table 2).

### Synthesis of results

**Fig. 2:**
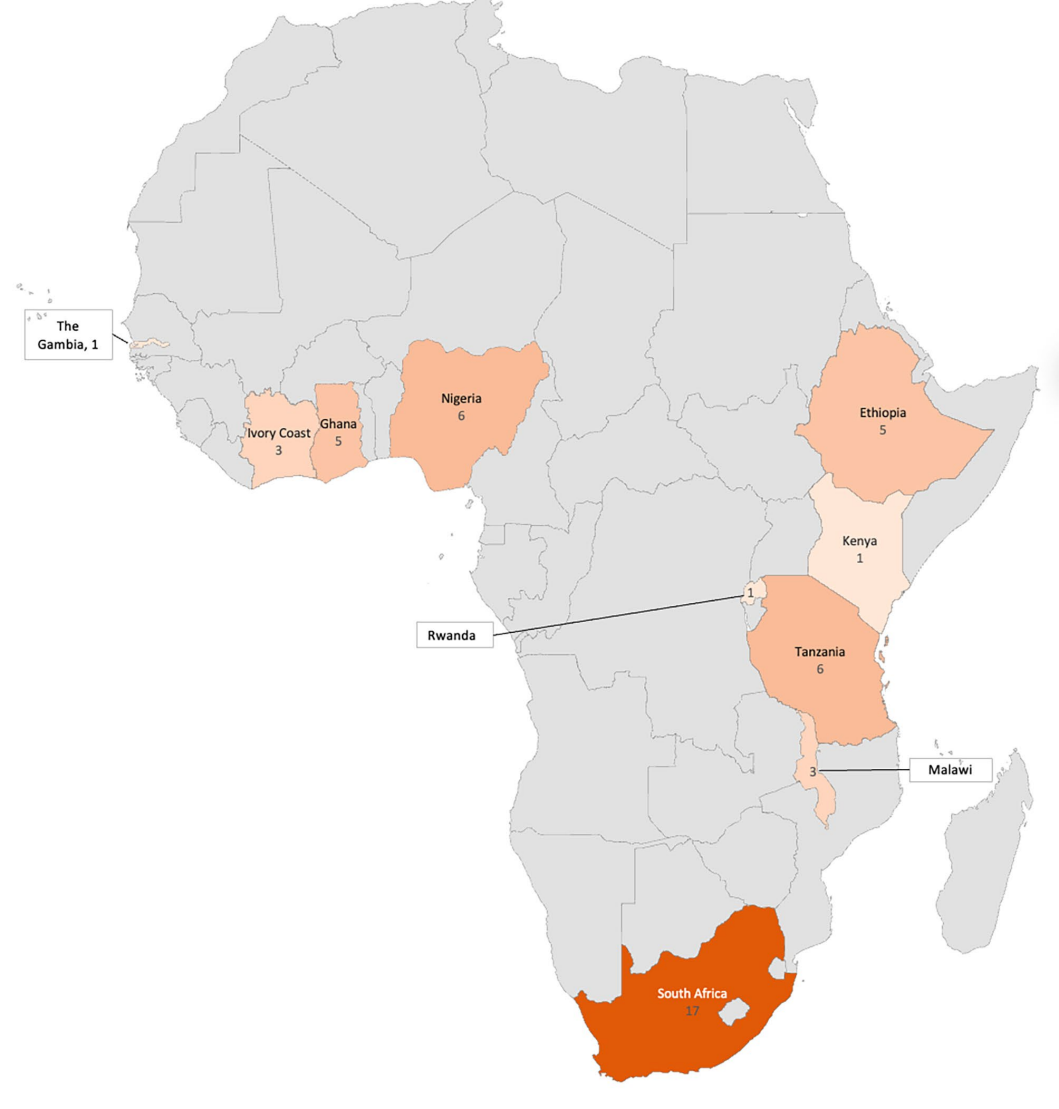
Frequency of studies on pregnancy-related anxiety by country

Research on pregnancy-related anxiety in SSA was conducted in 10 different countries (Table 4; Fig. 2). The most frequent locations for the studies were South Africa (11 studies); Tanzania, Ethiopia, and Nigeria (five studies each); and Ghana (four studies) (Table 4). Each use of a scale in each study was counted as one data point and extrapolated into a heat map (Fig. 2). If a study used multiple scales, it was counted as multiple points on the heat map (i.e., a study that used the Generalized Anxiety Disorder 7-item scale (GAD-7) and the EPDS would count for two points on the heat map).

The most-used tools to evaluate pregnancy-related mental health in SSA were the GAD-7 (seven uses), State-Trait Anxiety Inventory (STAI; five uses), and Self-Reporting Questionnaire 20 (SRQ-20; five uses) (Table 3).

The 35 studies included in this review yielded 24 different tools to evaluate pregnancy-related anxiety in SSA. Nineteen of the 24 tools (79%) used Likert scales for their question type, four (17%) used yes/no questions, and one (4%) used ranking on a number line. Importantly, only seven of the 24 tools were developed to be used specifically in pregnant populations. Only two tools were developed specifically for use in SSA—the 4-item Screening Tool and the Risk Factor Assessment (RFA). These were both developed in South Africa, with the intention of being applied there [16, 17]. Three other tools have been adapted for use in SSA: the Pregnancy-related Anxiety Scale (PrAS), the Pregnancy-Related Anxiety Questionnaire (PRAQ), and the Tilburg Pregnancy Distress Scale (TPDS) [9]. Studies included in this review were published between 1994 and 2022, with 74% (26/35) published in or after 2015, suggesting an increasing focus on pregnancy-related anxiety in SSA in recent years.

## Discussion

Few systematic reviews have described the screening tools used to evaluate pregnancy-related anxiety, and to our knowledge this review is the first to examine the use of these tools specifically in SSA. Three key themes emerged from this review: (1) the use of pregnancy-specific versus general anxiety scales; (2) the cultural relevance of screening tools specifically designed for LMICs—particularly SSA; and (3) the challenges of implementing these tools in these regions.

Regarding themes 1 and 2, of the 24 scales evaluated, only seven were developed specifically for pregnancy-related anxiety. Of these, only five were designed with LMIC contexts in mind: the RFA, the 4-Item Screening Tool, the PRAQ, the PrAS, and the TPDS [12, 21, 22]. Despite the relevance of these tools, the most frequently used tools in SSA were general anxiety scales, such as the GAD-7, the STAI, and the SRQ-20—none of which are pregnancy-specific. The frequent use of these general scales raises questions about their appropriateness in assessing pregnancy-related anxiety due to the significant clinical distinctions between pregnancy-related and general anxiety. Pregnancy introduces a unique set of stressors—financial, social, and health-related—that are not typically assessed by general anxiety scales [2, 12]. The GAD-7 has been validated for use in several LMICs, such as Zimbabwe and Ghana, but its lack of pregnancy-specific focus may limit its efficacy in evaluating pregnancy-related anxiety [55]. Nonetheless, a recent study in Peru did show that the GAD-7 may have promise in evaluating pregnancy-related anxiety; however, this requires further investigation [56]. The SRQ-20, though developed by the World Health Organization for use in LMICs, similarly lacks a pregnancy-specific focus [57]. The STAI, despite its widespread use, has limited validation in LMICs, with Malaysia being the sole LMIC in which it is validated [58, 59]. These findings underscore the importance of expanding the use and validation of pregnancy-specific scales in LMICs to ensure accurate assessment of pregnancy-related anxiety.

For theme 3, many obstacles remain when applying pregnancy-related anxiety screening tools to SSA contexts, as 19 of the 24 tools evaluated were developed for use in high-income countries. Cultural and economic stressors can be a source of anxiety for pregnant women, and scales developed in high-income countries may not adequately capture these factors and how they relate to pregnancy [10]. The practical challenges of implementing these tools—such as language barriers, difficulties in translating scales, time and resources needed to validate a screening tool in a new population [60], and the complexity of Likert scales—pose significant obstacles to their effective use in resource-limited settings.

### Strengths and limitations

This study was limited by three main factors. First, the exclusion of non-English language studies may have led to the omission of relevant research, particularly from French- and Portuguese-speaking countries in SSA. This limitation could introduce a bias in our findings, as it may not fully represent the diversity of experiences and practices across SSA. It also prevented the use of databases that may have been relevant to our search such as Africa Wide, limiting the generalizability of our findings. Second, while we included studies that utilized scales validated for use in LMICs, these validations were often limited to specific countries or regions, which may limit their generalizability to other LMIC contexts. Given the diverse cultural and socioeconomic environments within SSA, it is crucial to refrain from characterizing SSA as a monolith, and we must consider that a tool validated in one country or population may not be appropriate for another within SSA. Additionally, external factors such as political shifts or climate change may necessitate the reappraisal and revalidation of these scales over time. Third, our review included a number of studies that utilized general anxiety scales in their aim to evaluate pregnancy-related anxiety. These scales typically included a limited number of questions related to worries of pregnancy and may not fully capture the unique symptomatology and trajectory of anxiety during pregnancy [61].

## Conclusions

This scoping review sheds light on the current state of tools used to evaluate pregnancy-related anxiety in SSA and underscores the critical need for contextually appropriate tools that account for the unique stressors faced by pregnant women in these settings. Future research should prioritize tool development and validation, ensuring their practicality and ease of use in resource-constrained environments. This includes adapting tools to account for language barriers, simplifying scales for varying literacy, and ensuring compatibility with existing healthcare systems. Addressing these gaps may allow for future studies that track the course of pregnancy-related anxiety in SSA longitudinally, providing evidence to inform targeted interventions and policy decisions, and ultimately improving the mental health outcomes of pregnant women in these regions.

## Data Availability

No datasets were generated or analysed during the current study.

## Abbreviations

LMIC: Low- or middle-income country
SSA: Sub-Saharan Africa
PRISMA: Preferred Reporting Items for Systematic Reviews and Meta-Analysis
EPDS: Edinburgh Postnatal Depression Scale
GAD-7: Generalized Anxiety Disorder 7
STAI: State-Trait Anxiety Inventory
SRQ-20: Self-Reporting Questionnaire 20
PRAQ: Pregnancy Related Anxiety Questionnaire
PRAS-r: Pregnancy-related Anxiety Scale-revised
W-DEQ-A: Wijma Delivery Expectancy/Experience Questionnaire
RFA: Risk Factor Assessment
TPDS: Tilburg Pregnancy Distress Scale
SAS: Zung Self-rating Anxiety Scale
HADS: Hospital Anxiety and Depression Scale
WHODAS 2.0: World Health Organization Disability Assessment Schedule 2.0
K10: Kessler Psychological Distress Scale
GAD-2: Generalized Anxiety Disorder 2
BSI-18: Brief Symptoms Index
BAI: Beck Anxiety Inventory
PSS: Perceived Stress Scale
DASS-21: Depression Anxiety Stress Scale-21
